# Supplementary material for: Polarization-driven reversible actuation in a photo-responsive polymer composite
Source: Nat Commun. 2023 Oct 27;14:6843. doi: 10.1038/s41467-023-42590-y (PMC10611746; doi:10.1038/s41467-023-42590-y)
Supplement: Supplementary file 1 — Supplementary Information [file 41467_2023_42590_MOESM1_ESM.pdf]

Supplementary Information

Polarization-driven reversible actuation in a photoresponsive polymer composite

David Urban[1,2], Niccolò Marcucci[2], Christoph Hubertus Wölfle[3], Jan Torgersen[3], Dag Roar Hjelme[1] and Emiliano Descrovi[2]

Corresponding Author
emiliano.descrovi@polito.it

[1] Department of Electronic Systems, Norwegian University of Science and Technology, O.S. Bragstads plass 2b, 7034 Trondheim, Norway

[2] Dipartimento di Scienza Applicata e Tecnologia, Politecnico di Torino, Corso Duca degli Abruzzi 24, 10129, Torino, Italy

[3] Institute of Materials Science, Department of Materials Engineering, TUM School of Engineering and Design, Technical University of Munich, Boltzmannstraße 15, 85748 Garching, Germany

SUPPLEMENTARY FIGURES

Supplementary Figure 1 - Molecular structure, weight, and absorption spectrum of pDR1m-co-mma

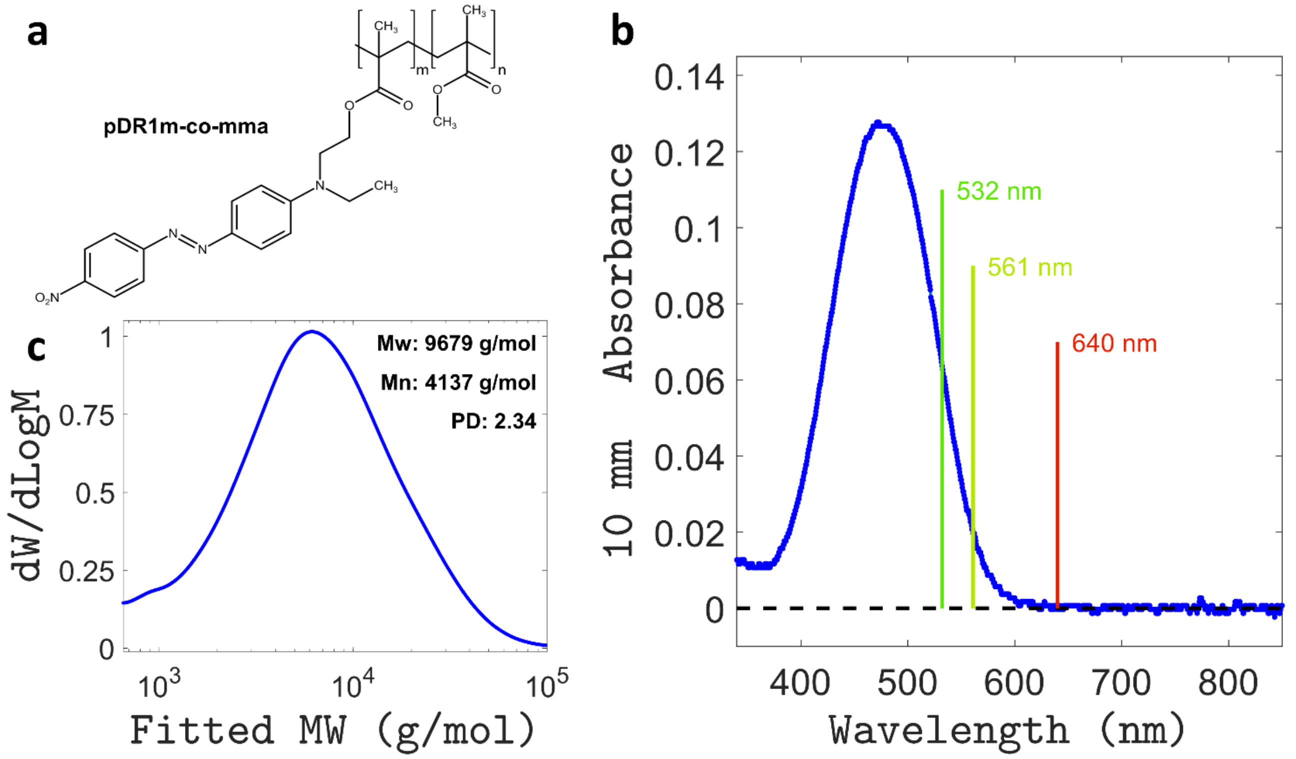

Supplementary Figure 1: (a) Molecular structure of poly [(methyl methacrylate)-co-(Disperse Red 1 methacrylate)] (pDR1m-co-mma). The approximate ratio of DR1 chromophores (m) was indicated as ~15 mol% by the manufacturer. (b) Absorption spectrum of pDR1m-co-mma measured at room temperature and dissolved at 0.0025 mg/mL in acetone. Vertical lines indicate the wavelengths used in this work. 640 nm: planar substrates reading mode. 561 nm planar substrates writing mode. 532 nm: 3D actuators writing mode. The spectrum mainly refers to the absorption by DR1 molecules in the trans isomeric state. (c) Normalized molecular weight distribution obtained by gel permeation chromatography (Agilent Technologies LC 1260) of the polymer dissolved in Tetrahydrofuran (THF) at room temperature, using a polystyrene standard. Inset: Values obtained for weight/number average molecular weight (Mw/Mn) and polydispersity index (PD).

Supplementary Figure 2 - Propagation of displacement outside the illuminated area

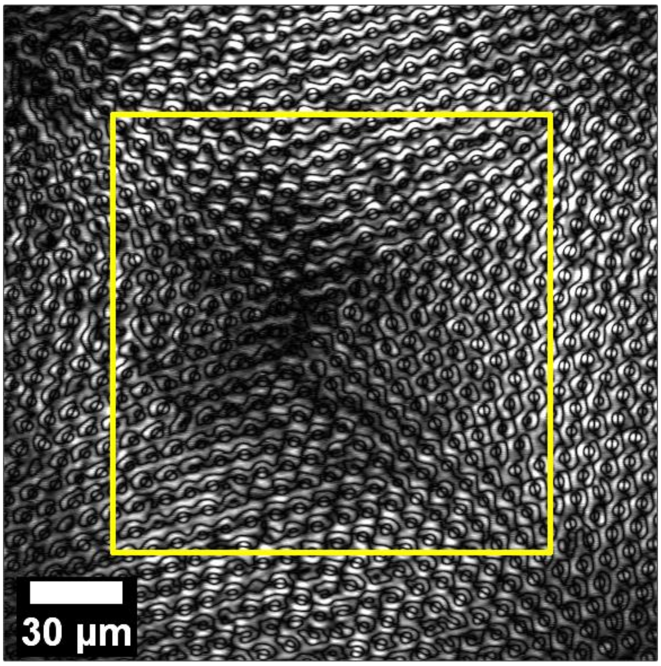

Supplementary Figure 2: Overview differential image of planar substrate before and after irradiation with linear horizontal polarization, qualitatively showing the amplitude of local displacement. Some displacement is seen to extend outside the region of irradiation (yellow frame), accommodating the deformation of the latter. Note that while displacement can be seen to occur in both x- and y directions (and is null in the center indicating absence of translation), the direction of displacement can hardly be discerned on such a plot. For directionality of deformation, see Supplementary Movie 1 (direct space) and/or Figure 2c,d (Fourier space).

Supplementary Figure 3 - Wrinkles on suspended AZO:SEBS film upon irradiation with linear horizontal polarization

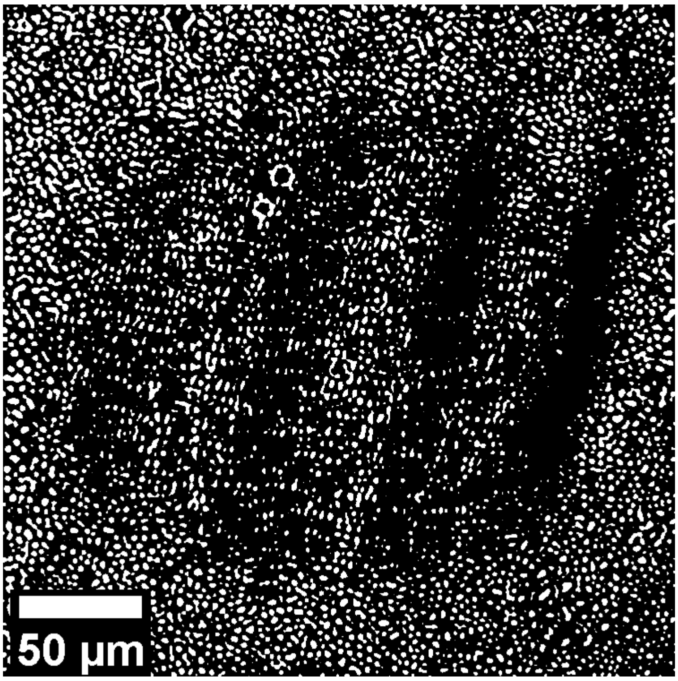

Supplementary Figure 3: Wrinkles upon irradiation with linear horizontal polarization: zoomed-in version of frame 58 of Supplementary Movie 2. Wrinkles have been highlighted through increased contrast and multiple iterations of local background subtraction and Gaussian blurring.

Supplementary Figure 4 – Cumulative sequential deformation of pDR1m micropillars

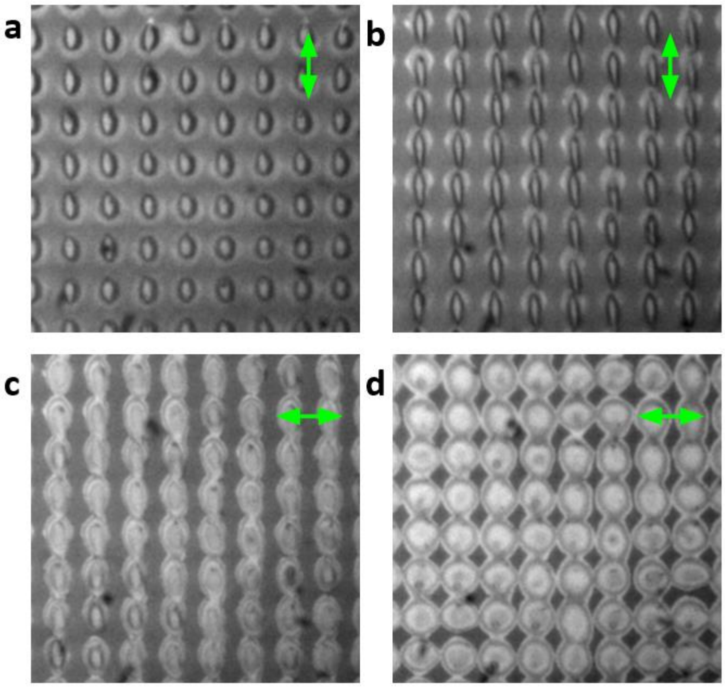

Supplementary Figure 4: (a,b) Early/final state of elongation during irradiation with linearly vertically polarized light. (c,d) Early/final state of deformation when subsequently illuminating the same pillars with perpendicular horizontal polarization. Green Arrow: Direction of polarization.

Supplementary Figure 5 – Light-induced deformation on a pure pDR1m-co-mma film

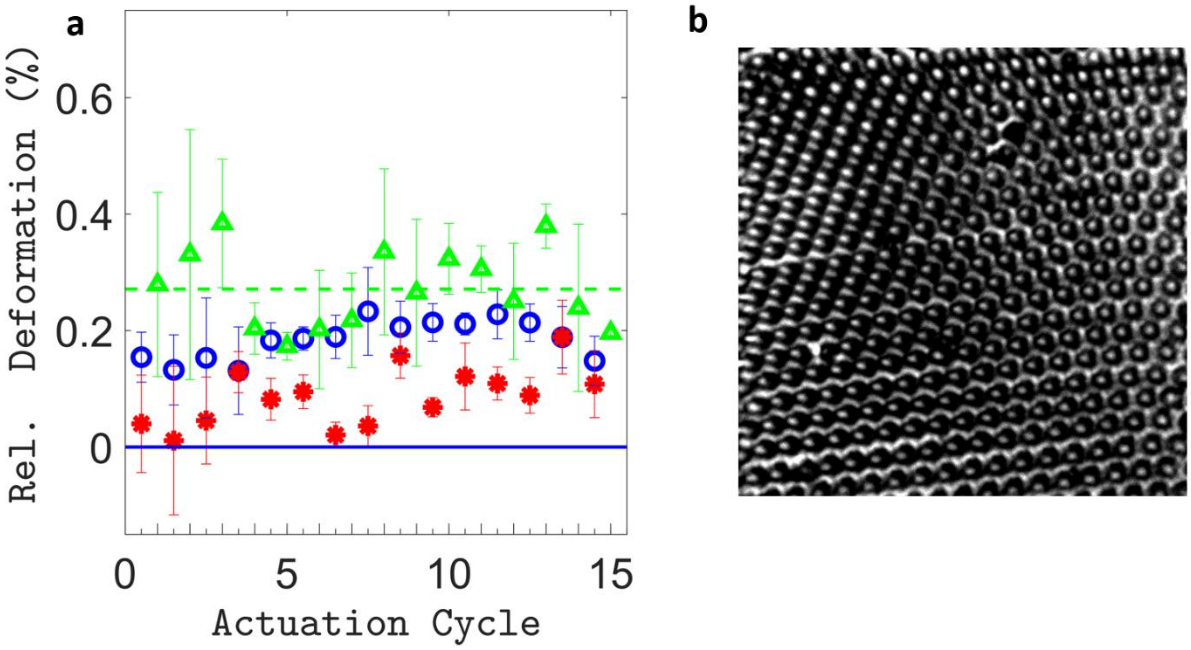

Supplementary Figure 5: Analysis of deformations on a 33.8 x 33.8 µm^2 area of pure azopolymer pDR1m-co-mma patterned with 1 µm spaced, 1 µm wide pillars, illuminated at intensity I = 5.02 W·cm^{-2}. (a) Graph showing relative horizontal elongation strain ε_1 per X-POL step (blue circles), absolute value of relative vertical compression strain $|\varepsilon_2|$ per X-POL step (red asterisks), and relative area expansion δA_{cycle} per full illumination cycle (green triangles). Parameters are shown for the 15 first illumination cycles only, due to gradual degradation of the pillar lattice by cumulative deformations as exemplified in (b), showing the direct space image after 20 illumination cycles (see also Supplementary Movie 3). Error bars: sample standard deviation for 3 acquisitions on different areas.

Supplementary Figure 6 - FE Modelling Details

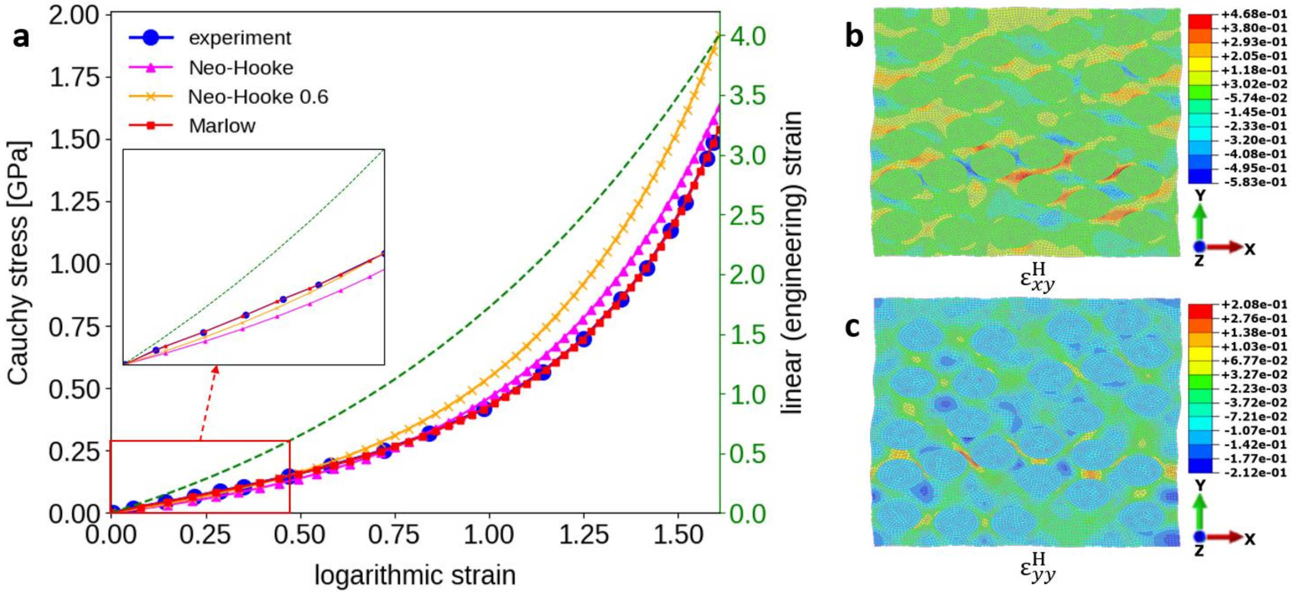

Supplementary Figure 6: (a) Fitting of the tensile testing data from literature (Kollosche et. al, 2010) with three models: Marlow model, Neo-Hookean model and Neo-Hookean model with up to 60% strain input data**.** Green dashed line: help line for transferring logarithmic strain into linear engineering strain (right vertical axis). Inset: Zoom-in on small strain region. (b,c) Simulated strain fields' off-diagonal logarithmic (Hencky) strain $\varepsilon^{\mathrm{H}}_{xy}$ and logarithmic normal strain $\varepsilon^{\mathrm{H}}_{yy}$ components around the elliptically deformed azo-domains.

Supplementary Figure 7 - Influence of irradiation parameters on AZO:SEBS film deformation

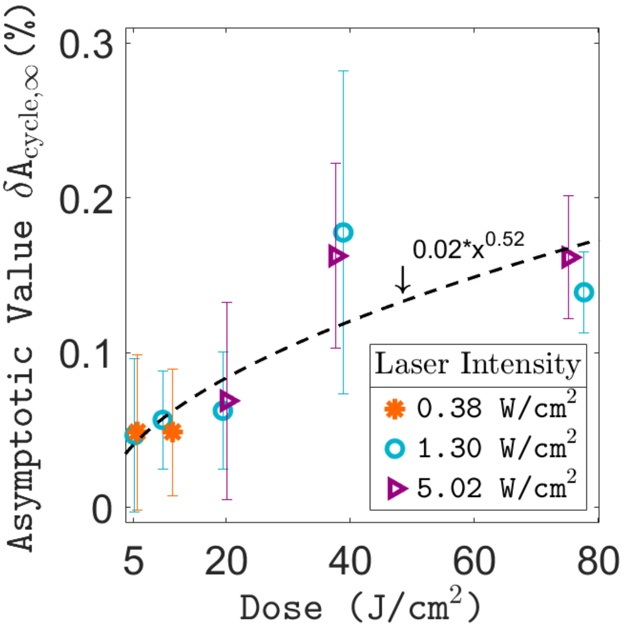

Supplementary Figure 7: Plot showing the asymptotic value of the relative area expansion $\delta A_{\mathrm{cycle},\infty}$ per illumination cycle as a function of applied dose, using the same measurements as in Figure 4. Error bars are three times the standard error of the mean (s.e.m.), as detailed in the Methods section.

Supplementary Figure 8 – Deformation parameters in several illumination conditions

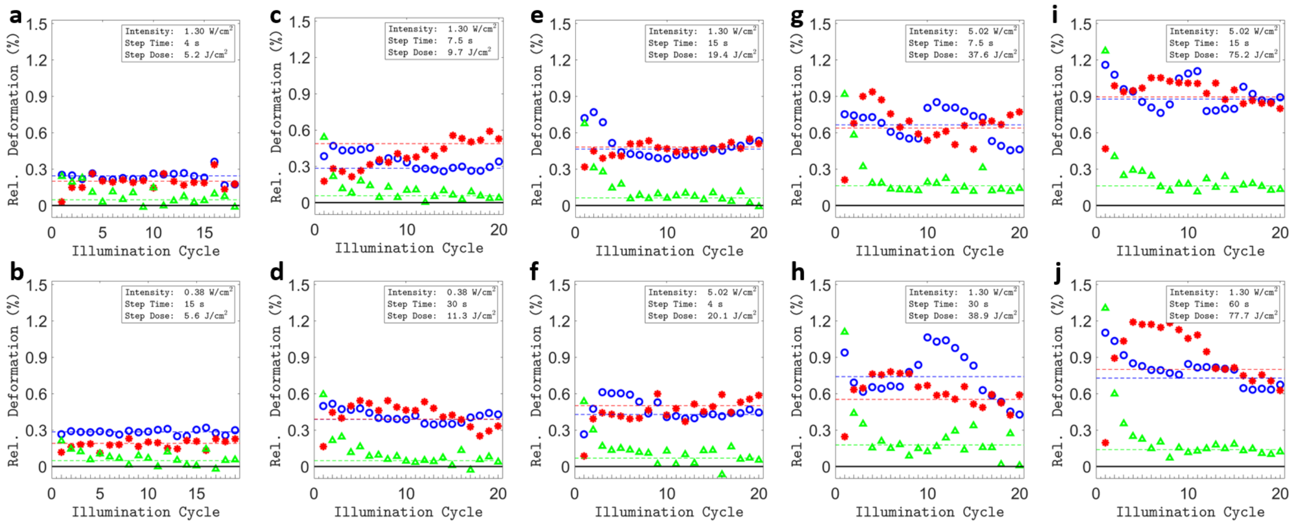

Supplementary Figure 8: 20 illumination cycles on areas of sample 1, using different laser powers and doses. (a-j) Graphs showing relative horizontal elongation strain ε_1 per X-POL step (blue circles), absolute value of relative vertical compression strain $|\varepsilon_2|$ per X-POL step (red asterisks), and relative area expansion per full illumination cycle (green triangles), for increasing value of the dose per illumination step. Helplines visualize the asymptotic values used in Figure 4 and Supplementary Figure 7 and are based on the mean of the last 10 respective datapoints.

Supplementary Figure 9 – Azopolymer domains in samples with different solvent content

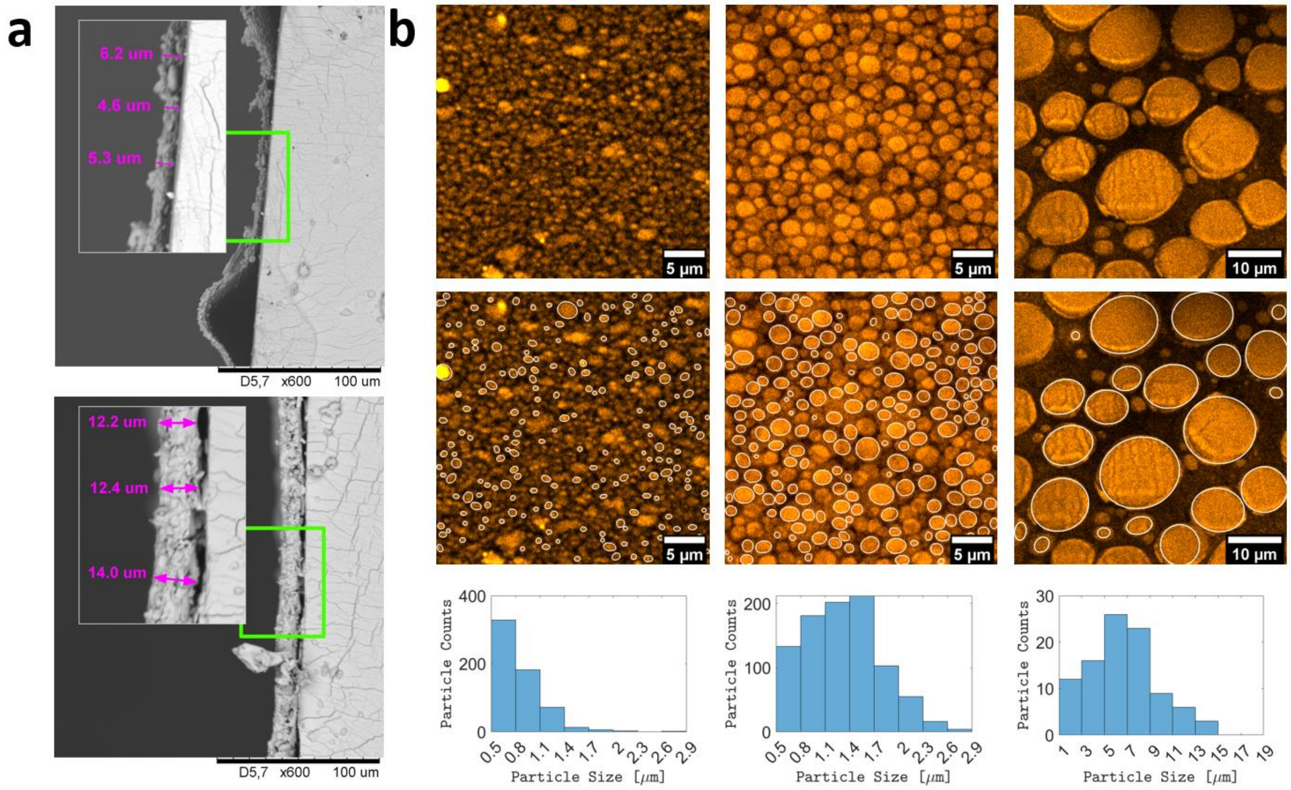

Supplementary Figure 9: (a) Examples of SEM cross-section images of different samples. Top-bottom: sample 2-3, inset: zoom-in on thickness measurement areas. (b) Examples of confocal images used for characterization of the largest aggregate sizes. Left to right: sample 1-3. Histograms show particle sizes summed over 5 similar images from distinct areas for each sample.

Supplementary Figure 10 - Sample 4 fine structure.

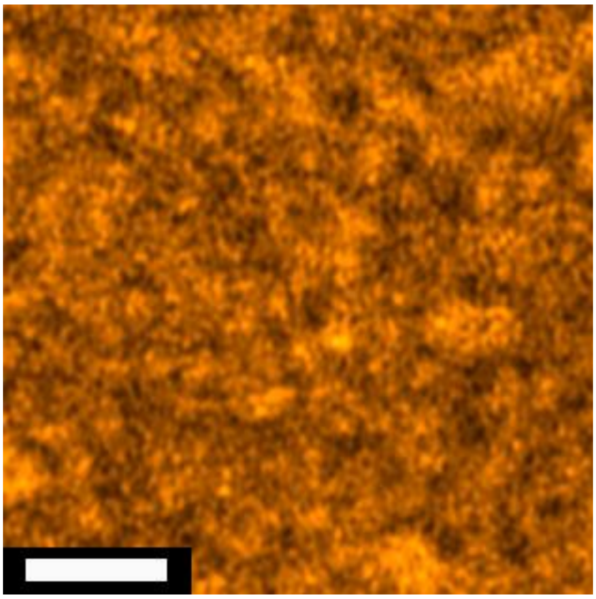

Supplementary Figure 10: Confocal image of interstitial area in-between large aggregates on sample 4. Scale bar: 3 µm.

Supplementary Figure 11 – Deformation of AZO:SEBS film with larger azopolymer domains

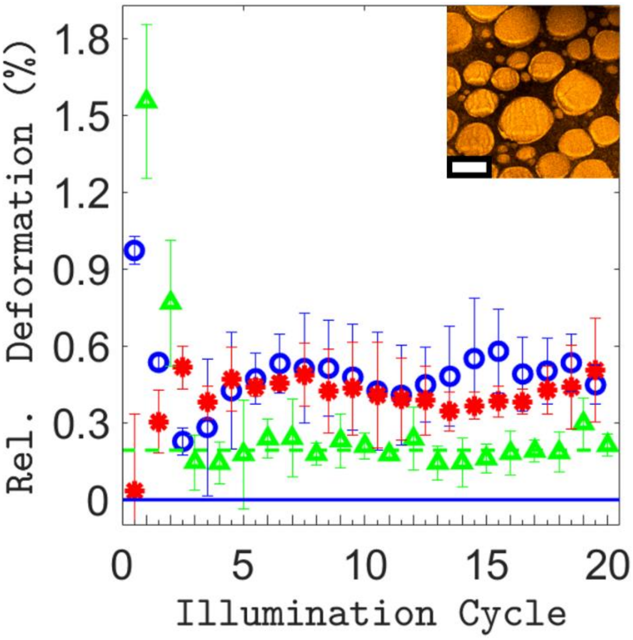

Supplementary Figure 11: Plot of fitted parameters for 20 illumination cycles for Sample 3. The graph shows relative horizontal elongation strain ε_1 per X-POL step (blue circles), absolute value of relative vertical compression strain $|\varepsilon_2|$ per X-POL step (red asterisks), and relative area expansion δA_{cycle} per full illumination cycle (green triangles). Error bars: sample standard deviation for 3 acquisitions on different areas. Inset: confocal image of sample fine structure and aggregate size. Scale bar: 10 μm.

Supplementary Figure 12 - Deformation induced by alternating linear polarizations

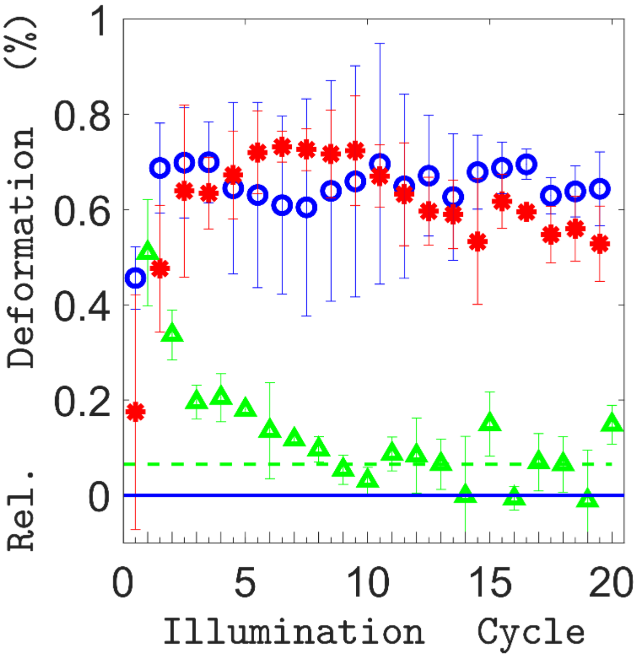

Supplementary Figure 12: Illumination cycles consist of horizontal linear followed by vertical linear polarization (instead of horizontal linear followed by circular polarization). Plot of fitted parameters, i.e., horizontal elongation strain ε_1 per X-POL step (blue circles), absolute value of relative vertical compression strain $|\varepsilon_2|$ per X-POL step (red asterisks) and relative area expansion δA_{cycle} per full illumination cycle (green triangles), for 20 illumination cycles on a sample similar to sample 1. Error bars: sample standard deviation for 3 acquisitions on different areas. Note that the characteristic feature of mostly reversible actuation (δA_{cycle} approaching a low asymptotic value) and area conservation for horizontally polarized illuminations in the switching regime (ε_1 comparable to ε_2) are still observed.

Supplementary Figure 13 - Effect of circularly polarized illumination on membranes

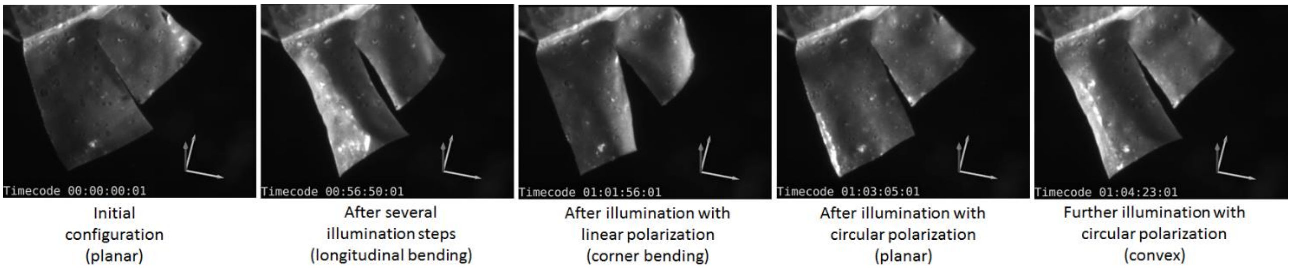

Supplementary Figure 13: From left to right: two co-planar cantilevers with similar widths and different lengths (initial state) are illuminated with a linear polarization transverse to the cantilever axis (“longitudinal bending” state). Then, the linear polarization is rotated, bringing the cantilevers into a "corner bending” state, similar to Figure 7c,d. The polarization is finally varied to circular. Both cantilevers revert bending, quickly reaching a planar configuration recalling the very initial state. With further illumination, a smooth convexity is observed to appear. This effect is likely to result from the adirectional in-plane expansion of azopolymer domains on top of the membrane.

Supplementary Figure 14 – Temperature on laser-irradiated AZO:SEBS films

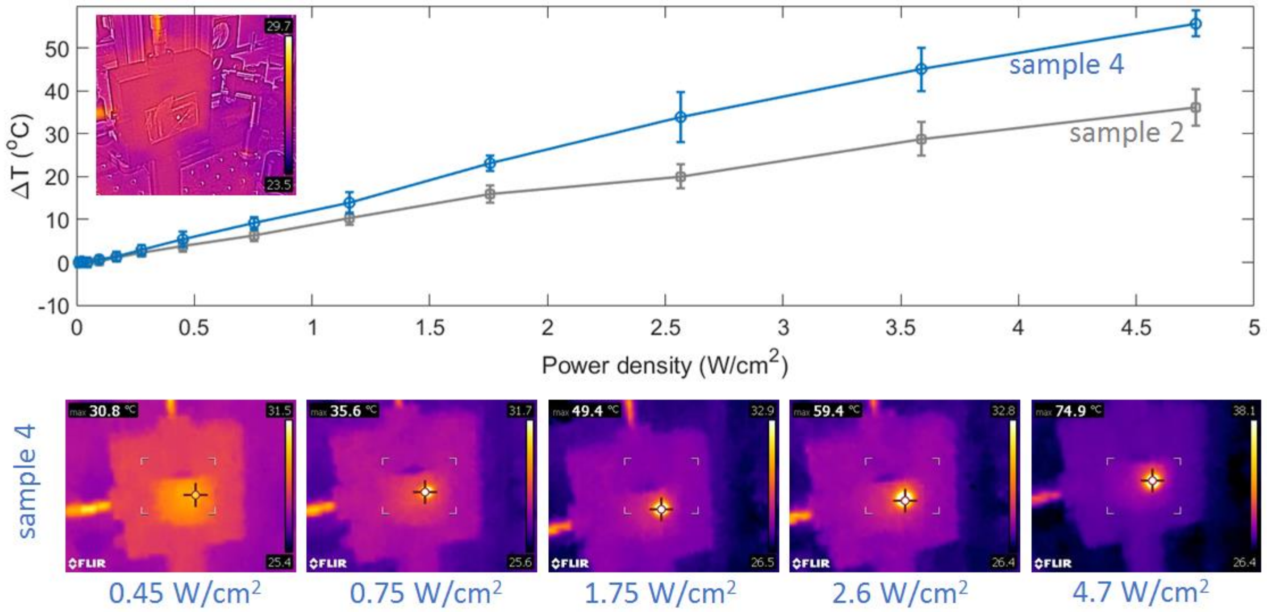

Supplementary Figure 14: Top line: measured temperature excess on azo-SEBS films on PDMS slabs. Sample type 2 and 4 are considered, wherein average azopolymer aggregate size and film thickness are smaller for sample 2 than sample 4. Bottom line: illustrative thermocamera images referred to sample 4 mounted on a 2-axis translational stage and being irradiated with a 532 nm wavelength CW laser at different power densities.

Supplementary Figure 15 – Deformation scheme of a pillar pattern on AZO:SEBS film

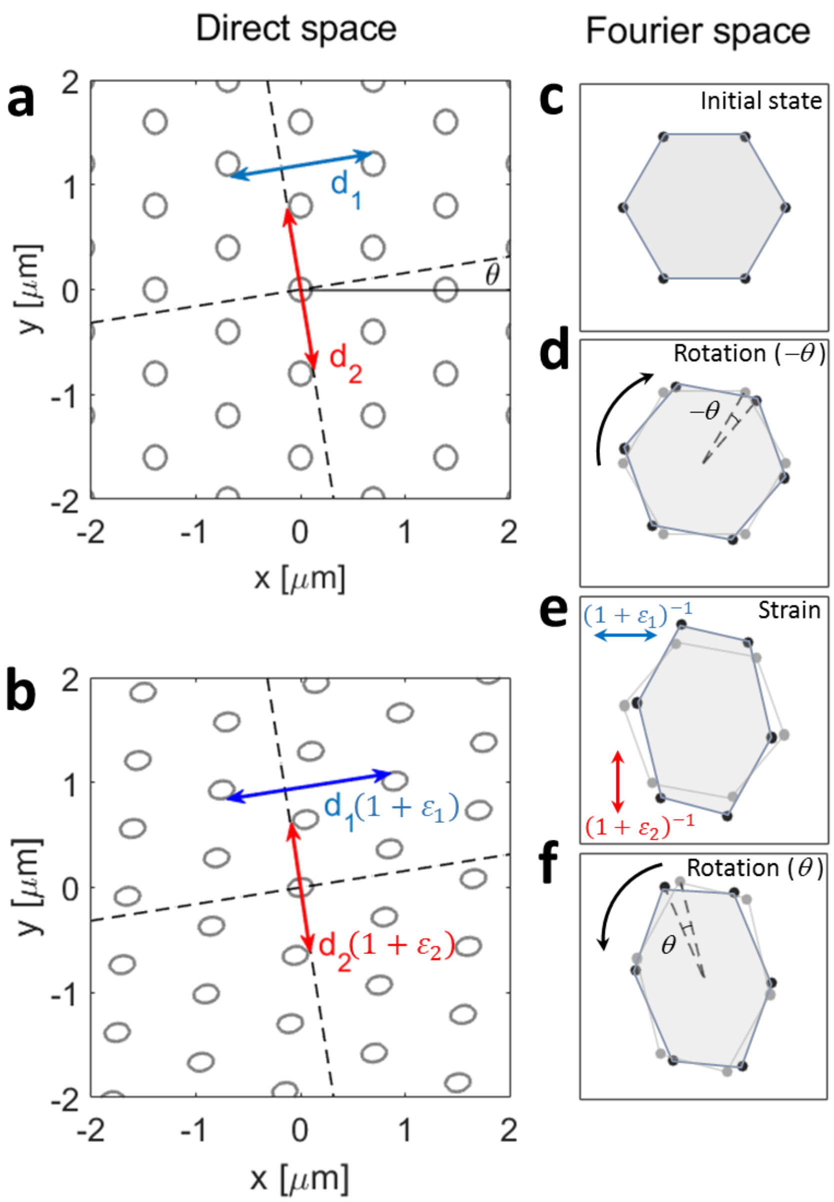

Supplementary Figure 15. (a) Illustrative hexagonal lattice in the direct space. Exemplary lengths d_1 and d_2 are taken along directions parallel and perpendicular to the incident polarization respectively, which is tilted by an angle θ with respect to the x-axis. (b) Photo-responsivity of the composite results into stretching ($\varepsilon_1>0$) and compression ($\varepsilon_2<0$) along said two orthogonal directions. (c) Reciprocal lattice cell in the initial state. (d) Reciprocal lattice cell after the first rotation by an angle $-\theta$. In this representation, the horizontal and vertical axis are aligned to the directions of compression and stretching in the Fourier space, respectively. (e) Lattice cell stretched and compressed along vertical and horizontal directions, respectively. (f) Deformed lattice cell in the final state, after a rotation back by an angle θ.

Supplementary Figure 16 – Impact of a two-dimensional computational model

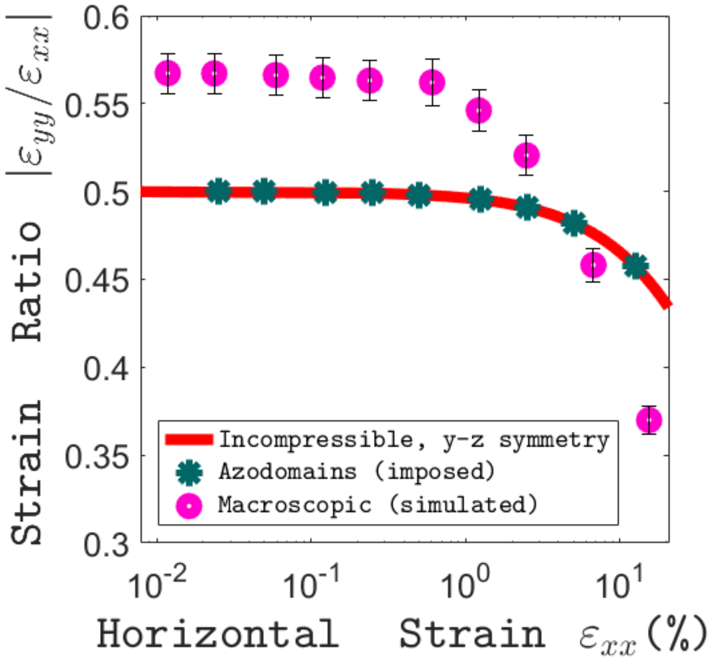

Supplementary Figure 16. Ratio of y-axis compression to x-axis elongation upon ellipsoid deformation, compared to the incompressible case with true axial symmetry around the x-axis (solid red line).

SUPPLEMENTARY NOTE 1 – First order approximations

The explicit computation of the switching tensor $\mathbf{F^3}$ proposed in Equation (3) can be obtained from $\mathbf{F^1}$ and $\mathbf{F^2}$, defined in Equation (2) in the article, via the following computation:

$$\mathbf{F^3} = \mathbf{F^1} \cdot (\mathbf{F^2})^{-1} = \begin{pmatrix} 1+\delta & 0 & 0 \\ 0 & 1-\frac{1}{2}\delta & 0 \\ 0 & 0 & 1-\frac{1}{2}\delta \end{pmatrix} \cdot \begin{pmatrix} \frac{1}{1+\frac{1}{4}\delta} & 0 & 0 \\ 0 & \frac{1}{1+\frac{1}{4}\delta} & 0 \\ 0 & 0 & \frac{1}{1-\frac{1}{2}\delta} \end{pmatrix}$$

$$= \begin{pmatrix} \frac{1+\delta}{1+\frac{1}{4}\delta} & 0 & 0 \\ 0 & \frac{1-\frac{1}{2}\delta}{1+\frac{1}{4}\delta} & 0 \\ 0 & 0 & \frac{1-\frac{1}{2}\delta}{1-\frac{1}{2}\delta} \end{pmatrix} \quad (6)$$

$$= \begin{pmatrix} (1+\delta)(1-\frac{1}{4}\delta+\cdots) & 0 & 0 \\ 0 & (1-\frac{1}{2}\delta)(1-\frac{1}{4}\delta+\cdots) & 0 \\ 0 & 0 & 1 \end{pmatrix}$$

$$= \begin{pmatrix} 1+\frac{3}{4}\delta+\cdots & 0 & 0 \\ 0 & 1-\frac{3}{4}\delta+\cdots & 0 \\ 0 & 0 & 1 \end{pmatrix} \approx \begin{pmatrix} 1+\frac{3}{4}\delta & 0 & 0 \\ 0 & 1-\frac{3}{4}\delta & 0 \\ 0 & 0 & 1 \end{pmatrix}$$

where the first line corresponds to inverting the individual elements to obtain the inverse of the diagonal tensor $\mathbf{F^2}$, the third line to a Taylor expansion writing elements up to the first order of δ and the fourth line to retaining only the first order elements.

Similarly, the tensor $\mathbf{F^{lin}}$ from Equation (5) can be shown to reduce to $\mathbf{F^1}$ from Equation (2), in a first order approximation:

$$\mathbf{F^{lin}} = \begin{pmatrix} a & 0 & 0 \\ 0 & 1/\sqrt{a} & 0 \\ 0 & 0 & 1/\sqrt{a} \end{pmatrix} = \begin{pmatrix} 1+\delta & 0 & 0 \\ 0 & 1/\sqrt{(1+\delta)} & 0 \\ 0 & 0 & 1/\sqrt{(1+\delta)} \end{pmatrix}$$

$$= \begin{pmatrix} 1+\delta & 0 & 0 \\ 0 & 1-\frac{1}{2}\delta+\cdots & 0 \\ 0 & 0 & 1-\frac{1}{2}\delta+\cdots \end{pmatrix} \approx \begin{pmatrix} 1+\delta & 0 & 0 \\ 0 & 1-\frac{1}{2}\delta & 0 \\ 0 & 0 & 1-\frac{1}{2}\delta \end{pmatrix} \tag{7}$$

$$= \mathbf{F^1}$$

where we have replaced a by $(1+\delta)$ and retained only the first order elements of δ in the Taylor expansion of each matrix element. In this framework δ can be associated to ε_1 (and -1/2 δ to ε_2), hence the Taylor expansion procedure can be seen as small strain approximation. A similar development can be done for $\mathbf{F^{circ}}$, reducing to $\mathbf{F^2}$ in a first order approximation. In that case, to enforce equal compression along the z-axis, one should set $1/\sqrt{a} = 1/b^2$ and therefore start the Taylor expansion with $b = a^{1/4} = (1+\delta)^{1/4}$, leading to:

$$\mathbf{F^{circ}} = \begin{pmatrix} b & 0 & 0 \\ 0 & b & 0 \\ 0 & 0 & 1/b^2 \end{pmatrix} = \begin{pmatrix} (1+\delta)^{1/4} & 0 & 0 \\ 0 & (1+\delta)^{1/4} & 0 \\ 0 & 0 & 1/(1+\delta)^{1/2} \end{pmatrix}$$

$$\approx \begin{pmatrix} 1+\frac{1}{4}\delta & 0 & 0 \\ 0 & 1+\frac{1}{4}\delta & 0 \\ 0 & 0 & 1-\frac{1}{2}\delta \end{pmatrix} = \mathbf{F^2} \tag{8}$$

SUPPLEMENTARY NOTE 2 – Accuracy of the 2D modelling approach

To assess the accuracy of the 2D modelling approach, one may compare it to a full 3D simulation of an isotropic representative volume of the azo-SEBS composite. In the 3D case, the axial symmetry around the x-axis, for uniaxial stretching along said axis by a factor a, should lead to equal lateral compression values for both perpendicular directions. Given the incompressibility assumption for all constituents of the material, the total simulation volume should be conserved as well, forcing the two lateral compression values to a factor $1/\sqrt{a}$ as apparent from the deformation gradient tensor for the incompressible deformation corresponding to uniaxial tensile loading, $\mathbf{F^{lin}}$, shown in Equation (5). In a small strain setting, one will then recover the Poisson ratio $-\varepsilon_{yy}/\varepsilon_{xx} = 0.5$, whilst for larger strains, incompressibility will dictate that $-\varepsilon_{yy}/\varepsilon_{xx} = \frac{a^{-0.5}-1}{a-1}$, a line which is plotted in the Supplementary Figure 16 (red line).

Whilst the imposed azo-domain deformations fulfill that condition by definition, the macroscopic average strains computed for the entire simulation domain deviate slightly from that ratio, by about 10 % averaged over the 9 different random geometries we studied. In practice, that means that the simulated y-axis compression of the overall material is slightly stronger than expected, at the expense of the not explicitly computed compression along the z-axis. Roughly speaking, the 2D simulation makes it easier for the composite to get pushed out along the z-axis than what would be expected for an isotropic 3D material.

This effect is reflected in Figure 3c, where the transmission in the simulation using $\mathbf{F^{lin}}$ is slightly higher along the y-axis (T_{yy}^{lin}) than along the x-axis (T_{xx}^{lin}). Furthermore, the normal transmission factors for the in-plane expansion of azo-domains upon applying $\mathbf{F^{circ}}$, i.e., T_{xx}^{circ} and T_{yy}^{circ}, are the lowest. This is likely again due to the 2D simulation permitting more material to be pushed out along the z-axis during this in-plane expansion than what would be the case in a 3D isotropic material. Finally, it should be stressed that the actual experimental samples lie somewhere between these 2D

and 3D cases, since the largest domain/aggregates are of comparable size to the photo-responsive layer's thickness (see Table 1).
