# Supplementary material for: Polarization-driven reversible actuation in a photo-responsive polymer composite
Source: Nat Commun. 2023 Oct 27;14:6843. doi: 10.1038/s41467-023-42590-y (PMC10611746; doi:10.1038/s41467-023-42590-y)
Supplement: Supplementary file 3 — Description of additional supplementary files [file 41467_2023_42590_MOESM3_ESM.pdf]

Description of additional supplementary files

Supplementary Movie 1 : Sequence of frames based on transmission images of a 33.8 x 33.8 µm2 area of azo-SEBS composite, decorated with 1 µm spaced, 1 µm wide pillars, and residing on a PDMS slab. Frames are acquired before and after illumination steps with alternating horizontal linear and circular polarization of the laser beam. Wavelength λ = 561 nm, intensity I = 5.02 W·cm-2 , illumination step time t = 15 s.

Supplementary Movie 2 : Sequence of frames showing transmission images of a thin (thickness: 5 µm) suspended azo-SEBS membrane whose center region is illuminated by a laser beam (diameter: 200 µm). Frames are acquired before and after illumination steps with alternating horizontal linear and circular polarization of the laser beam. Wavelength λ = 532 nm, intensity I = 2.6 W·cm-2 , illumination step time t = 15 s.

Supplementary Movie 3 : Sequence of frames based on scattered laser beam images of a 33.8 x 33.8 µm2 area of pure azopolymer, decorated with 1 µm spaced, 1 µm wide pillars, and residing on a PDMS slab. Frames are acquired before and after illumination steps with alternating horizontal linear and circular polarization of the laser beam. Wavelength λ = 561 nm, intensity I = 5.02 W·cm-2 , illumination step time t = 15 s.

Supplementary Movie 4 : Movie showing the smooth rotation of the bending axis of a circular azo-SEBS membrane patch placed on a needle, exposed to a λ = 532 nm laser beam impinging on the sample from the top (zaxis), with an intensity I = 1 W·cm-2 , and with continuously rotating polarization (green arrow).

Supplementary Movie 5 : Movie showing an azo-SEBS membrane patch undergoing deformation sequences during top illumination with a laser beam (λ = 532 nm, I = 1 W·cm-2). Polarization (green arrow) is altered in discrete steps, which are repeated in water, in air, and in water again.

Supplementary Movie 6 : Trapezoidal azo-SEBS membrane glued to a rigid boundary constraint undergoing deformations during top illumination (incidence direction: white arrow, λ = 532 nm, I = 1 W·cm-2) with polarization (green arrow) varied both in discrete steps and continuously.
